# Modelling forest carbon stock changes as affected by harvest and natural disturbances. II. EU-level analysis

**DOI:** 10.1186/s13021-016-0059-4

**Published:** 2016-08-26

**Authors:** Roberto Pilli, Giacomo Grassi, Werner A. Kurz, Jose V. Moris, Raúl Abad Viñas

**Affiliations:** 1 European Commission, Joint Research Centre, Directorate D – Sustainable Resources - Bio-Economy Unit, Via E. Fermi 2749, 21027 Ispra, VA Italy; 2 Natural Resources Canada, Canadian Forest Service, Victoria, BC V8Z 1M5 Canada; 3 Department of Agriculture, Forest and Food Sciences, University of Torino, Via Leonardo Da Vinci 44, 10095 Grugliasco, TO Italy

**Keywords:** EU, Carbon Budget Model, Forest management, Afforestation, Deforestation, Harvest, Natural disturbances, GHGI

## Abstract

**Background:**

Forests and the forest sector may play an important role in mitigating climate change. The Paris Agreement and the recent legislative proposal to include the land use sector in the EU 2030 climate targets reflect this expectation. However, greater confidence on estimates from national greenhouse gas inventories (GHGI) and more comprehensive analyses of mitigation options are needed to seize this mitigation potential. The aim of this paper is to provide a tool at EU level for verifying the EU GHGI and for simulating specific policy and forest management scenarios. Therefore, the Carbon Budget Model (CBM) was applied for an integrated assessment of the EU forest carbon (C) balance from 2000 to 2012, including: (i) estimates of the C stock and net CO_2_ emissions for forest management (FM), afforestation/reforestation (AR) and deforestation (D), covering carbon in both the forest and the harvest wood product (HWP) pools; (ii) an overall analysis of the C dynamics associated with harvest and natural disturbances (mainly storms and fires); (iii) a comparison of our estimates with the data reported in the EU GHGI.

**Results:**

Overall, the average annual FM sink (−365 Mt CO_2_ year^−1^) estimated by the CBM in the period 2000–2012 corresponds to about 7 % of total GHG emissions at the EU level for the same period (excluding land use, land-use change and forestry). The HWP pool sink (−44 Mt CO_2_ year^−1^) contributes an additional 1 %. Emissions from D (about 33 Mt CO_2_ year^−1^) are more than compensated by the sink in AR (about 43 Mt CO_2_ year^−1^ over the period). For FM, the estimates from the CBM were about 8 % lower than the EU GHGI, a value well within the typical uncertainty range of the EU forest sink estimates. For AR and D the match with the EU GHGI was nearly perfect (difference <±2 % in the period 2008–2012). Our analysis on harvest and natural disturbances shows that: (i) the impact of harvest is much greater than natural disturbances but, because of salvage logging (often very relevant), the impact of natural disturbances is often not easily distinguishable from the impact of harvest, and (ii) the impact of storms on the biomass C stock is 5–10 times greater than fires, but while storms cause only indirect emissions (i.e., a transfer of C from living biomass to dead organic matter), fires cause both direct and indirect emissions.

**Conclusions:**

This study presents the application of a consistent methodological approach, based on an inventory-based model, adapted to the forest management conditions of EU countries. The approach captures, with satisfactory detail, the C sink reported in the EU GHGI and the country-specific variability due to harvest, natural disturbances and land-use changes. To our knowledge, this is the most comprehensive study of its kind at EU level, i.e., including all the forest pools, HWP and natural disturbances, and a comparison with the EU GHGI. The results provide the basis for possible future policy-relevant applications of this model, e.g., as a tool to support GHGIs (e.g., on accounting for natural disturbances) and to verify the EU GHGI, and for the simulation of specific scenarios at EU level.

## Background

An effective role of forests in climate change mitigation requires a comprehensive assessment, from scientific and policy perspectives. From a scientific point of view, recent studies demonstrate the relevance of biophysical aspects of the forest-climate interactions, that may be important locally or in specific time frames [1]. Even if the recent paper by Naudts et al. [2], casting doubts on the role of European forests in mitigating climate change over the last centuries, there is increasing and largely consistent scientific evidence that forests in Europe are currently making a relevant and positive contribution to climate change mitigation (see [3, 4]). From a policy perspective, it is relevant to understand how this contribution may be efficiency translated into different mitigation options, including the sink in the forest, the sink outside the forest (in harvested wood products, HWP) and the use of wood for energy and material substitution [5]. Given the heterogeneity of the European forest system, assessing the specific regional circumstances, opportunities and challenges is fundamental [6]. At the same time, maximizing the sum of these mitigation options requires an integrated, dynamic modeling framework to quantify in a robust way the unavoidable trade-offs (e.g. between the forest sink and the bioenergy), which are often not appropriately considered [7]. Furthermore, for such a modeling framework to be directly policy-relevant, the policy context such as the rules for reporting and accounting emissions and removals from forests need to be taken into account.

In particular, the current rules under the Kyoto Protocol (KP) significantly changed for the second commitment period (CP2, 2013–2020) (see, [8–10]). According to these new rules, as reflected in the latest IPCC guidance [11]: (i) the reporting and accounting of forest management (FM, i.e., land in the forest land use category in 1989) are now mandatory (through a ‘forest reference level’), in addition to the already mandatory accounting of afforestation/reforestation and deforestation (AR and D, i.e., forest land-use changes since 1990); (ii) the accounting of FM shall include the carbon (C) stock changes in the HWP pool; and (iii) emissions and subsequent removals from natural disturbances may be excluded from the accounting under certain conditions. While some further change is foreseen under the proposed post-2020 EU regulation on land use and forestry [12], most of these rules are expected to continue (e.g. forest reference level, HWP, natural disturbances).

The greenhouse gas inventories (GHGIs) represent the basis to assess the compliance of any climate mitigation target. The GHGI of the EU, submitted annually to the United Nations Framework Convention on Climate Change (UNFCCC) and its KP, is the sum of the inventories of 28 Members States (MS), which include about 158 Mha of forests [13]. The species composition, the current and past management practices, the amount of natural disturbances, and the quality and type of information available on the forest resources, differ among countries. Moreover, conceptual and methodological differences in countries’ GHGIs produce discrepancies in the resulting estimates that are currently not entirely addressed and require further work to achieve reliable and consistent estimates throughout Europe [14, 15].

The complex EU forest sector can be represented using a process-based approach (e.g. [1, 16]) or using, for each country, empirical forest-inventory based models (e.g. [17]). Traditionally, process-based models have mainly been used to simulate the long-term evolution of forest C dynamics at large scales, including the potential effects of climate change [18], but they generally do not include a detailed analysis of forest management practices. Therefore, empirical models still remain the primary tool to simulate the detailed effects of different management options on short-term forest C dynamics [19, 20] at small to medium spatial scales (e.g. from forest stands to countries). When compiled from regional or country level scales to a continental scale, the empirical model results can be compared with the data produced through process-based approaches [21, 22], and can provide additional information on the main drivers of forest carbon dynamics at the EU scale. Different forest-inventory based models were used in the European context, to estimate the future forest C sink under different policies and management scenarios (i.e., [20]) or the impact of natural disturbances on the forest C stock [23] or the realizable potential supply of woody biomass [24]. None of these studies, however, considered in a comprehensive way the overall EU forest C sink consistently with the current international reporting and accounting regulations, i.e., including FM, AR and D, HWP and natural disturbances.

In a recent study, the Carbon Budget Model (CBM), developed by the Canadian Forest Service [25] was applied to 26 EU MSs to model the forest C dynamics from FM at the country level for the period 2000–2012, including the impact of the major natural disturbances [26]. In that study, after having validated the CBM results for a representative country, the country-specific results were evaluated against the individual 2014 GHGIs submitted to the UNFCCC by each EU MS. This evaluation is an essential pre-requisite to analyze the overall EU forest C balance and the level of confidence on the EU GHGI. Achieving this confidence is key to allow the forest sink to be included in the EU climate target [27]. The goal of this paper is to provide a tool for verifying the whole EU GHGI and for simulating specific policy and forest management scenarios at EU level. In particular, with the present paper, focused at the EU level and largely based on the same methodological assumptions used by Pilli et al. [26], we aim to: (i) estimate the C stock and the CO_2_ emissions and removals for FM, covering the carbon both in the forest pools (total living biomass, dead organic matter, mineral soil) and in HWP pool; (ii) estimate the CO_2_ emissions and removals for forest land-use changes (i.e., AR and D); (iii) provide an overall EU-level analysis of the impacts of harvest and natural disturbances (mainly storms and fires); and (iv) compare our estimates with the estimates reported in the EU GHGI and other continental-scale studies.

## Results and discussion

The aggregated results at the EU level for the forest-related activities defined by the KP are reported for FM (i.e., forest existing in 1989) AR and D (i.e., forest and land-use changes since 1990) in “Forest C stock (2008–2012)” section. Here, the results obtained using the CBM are compared with the data reported in the 2014 EU GHGI^1^ [13]. According to the EU GHGI, the two MSs not considered in this study, Cyprus and Malta, provide a negligible contribution to the EU forest sink (0.02 %).

The resulting forest C dynamics are described in “Forest C dynamics (2000–2012)” section. Here, the data are reported from an atmospheric perspective, where negative values represent a sink (CO_2_ removals) and positive values a source (CO_2_ emissions). Results cover only CO_2_ and exclude organic soils. Even if emissions from drained organic soils and non-CO_2_ emissions from forest fires may be relevant in some countries (e.g., [28, 29]), at the EU level they account for 5 and 2 % (in terms of CO_2_-eq.), respectively, of net annual forest sink [13]. In “Main drivers determining forest C dynamics” section, we discuss the main drivers determining the forest C sink dynamic, further distinguished between harvest (“Harvest” section) and natural disturbances (“Natural disturbances” section).

### Forest C stock (2008–2012)

The average C stock per hectare estimated for FM by the model for the period 2008–2012 (i.e., the First Commitment Period, CP1, of the KP), is equal to 142.3 Mg C ha^−1^ at the EU level, including 68.4, 19.3 and 54.5 Mg C ha^−1^, for living biomass, DOM and mineral soil, respectively (Table 1). According to Pilli et al. [30], the total C stock of HWP is about 1921 Tg C (average for the CP1), which compares the total C stock in living biomass of 9437 Tg C in this study.

**Table 1:** Average C stock estimated for FM by the CBM model for the period 2008–2012 (KP-CP1) at EU level

Average historical (2008–2012) C stock		DOM		Soil	Living biomass			Total ecosystem
		Deadw.	Litter		Abovegr.	Belowgr.	Tot Liv. Biom.	
C stock	Av. (Mg C ha^−1^)	10.8	8.5	54.5	56.0	12.4	68.4	142.3
	Total (Tg C)	1493	1177	7524	7720	1717	9437	19,632

Overall, our estimates on living biomass are in good agreement with the data reported by most of other reports or studies. The Global Forest Resource Assessment 2010 [31] reports for 2010 in Europe, excluding the Russian Federation but including some non-EU countries (i.e., for a total forest area that is about 8 Mha larger than the forest area considered by our study), an average C stock equal to 63.9, 18.6 and 96.6 Mg C ha^−1^, for the living biomass, dead wood plus litter and soil (including peat), respectively. The State of Europe’s Forests 2015 [32] reports for EU-28 and assuming a forest area slightly larger than our study (+9 %) a biomass C stock in 2010 equal to 58.8 Mg C ha^−1^.

Providing estimates for dead wood and litter C stock is difficult [33]. Verkerk et al. [34] applied the EFISCEN model to 24 EU MS (i.e., the same considered by our study, except Croatia and Greece) and estimated an average amount of deadwood of 12.3 t ha^−1^ in 2005. Assuming a C content of 0.5, this equals an average C stock of 6.1 t C ha^−1^, about 40 % lower than our estimate.

An accurate assessment of the soil C stock is also difficult due to the range of available model and inventory results [33, 35]. Our estimate of the total C stock in forest mineral soils (7524 Tg C) is intermediate between the values reported by other studies: about 5000 Tg C reported by Liski et al. [36] and 13,700 Tg C (including the O-layer) estimated by Goodale et al. [37]. Since the soil C stock is affected by both natural and anthropogenic factors [38, 39], comparing the average C stock estimated by our model, equal to 54.5 Mg C ha^−1^, with other studies is even more difficult. Shulp et al. [40] report, for the Netherlands, a mean C stock in mineral soil between 53 and 97 Mg C ha^−1^, with significant statistical differences mainly due to the species composition. The average C stock in mineral soil estimated by the last Italian National Forest Inventory (NFI) (concluded in 2009) varies between 68 and 96 Mg C ha^−1^, depending by the species composition [41], while the Swedish NFI estimated, for 2000, an average C stock for coniferous forest soil, equal to 73 ± 10 Mg C ha^−1^ [42].

### Forest C dynamics (2000–2012)

**Fig. 1:**
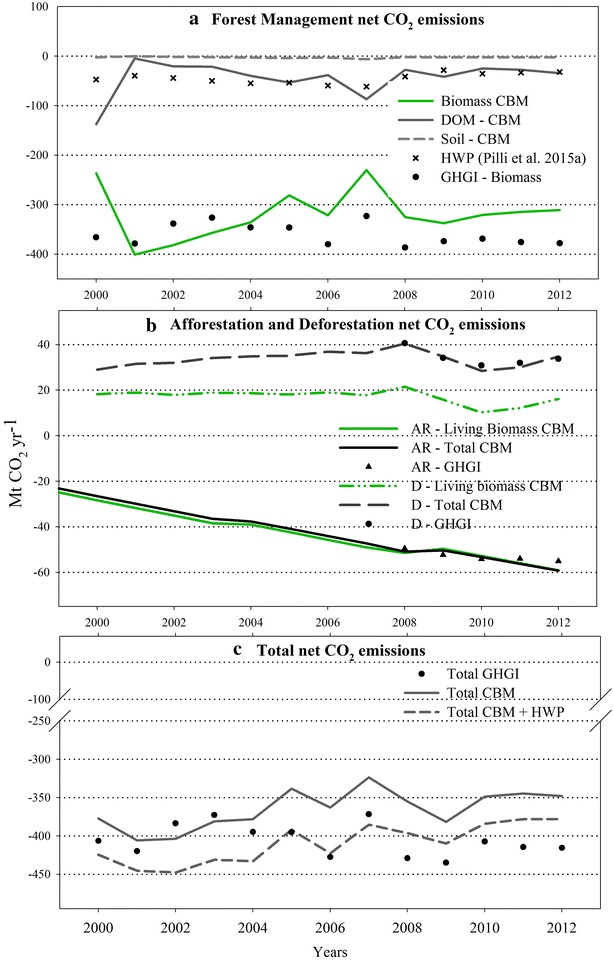
EU-level net CO_2_ emissions (in Mt CO_2_ year^−1^) for: **a** Forest management (FM), as estimated by the CBM for the living biomass, DOM and soil pools, by Pilli et al. [30] for the HWP pool and as reported for the living biomass pool in the EU GHGI (see [13] and the “Methods” for further details); **b** afforestation/reforestation (AR) and deforestation (D) since 1990, as estimated by the CBM for living biomass and all the pools (total) and as reported (all pools) in the KP CRF tables of the EU GHGI; **c** sum of FM, AR and D, as estimated for all the pools by the CBM (with and without HWP) and as reported in the EU GHGI (without HWP). Organic soils are always excluded from GHGIs, to allow a more consistent comparison with CBM. All the values are reported from an atmospheric perspective, i.e., negative values represent a sink and positive a source

The sum of the net CO_2_ removals from all land-use activities and carbon pools considered (FM + AR + D + HWP) is, on average, equal to −409 Mt CO_2_ year^−1^ between 2000 and 2012 (Fig. 1, panel c). This corresponds to about 8 % of the total GHG emissions in the EU for the same period (without LULUCF). This amount may be further distinguished between different land-use activities (FM, AR and D) and pools. About 90 % of the total C sink is due to FM (Fig. 1, panel a, including HWP), while AR (Fig. 1, panel b) contributes to the remaining 10 %, with an increasing fraction due to the ageing of the new forest area. Deforestation is a source by about 33 Mt CO_2_ year^−1^ between 2000 and 2012. About 80 % of the total C sink (after subtracting emissions from D) is due to the living biomass pool (70 % accounted as FM and 10 % as AR), 10 % is due to DOM (mainly accounted as FM) and the remaining 10 % is related to the HWP pool.

Within FM, the highest inter-annual variations (due to harvest and natural disturbances) were estimated for the living biomass pool, varying from −237 Mt CO_2_ year^−1^ in 2000 to −311 Mt CO_2_ year^−1^ in 2012 (Fig. 1, panel a). As expected, the DOM pool in the CBM has the opposite trend, because natural disturbances such as storms, fires and insect attacks transfer carbon from biomass to DOM pools (see for example 2000, 2005 and 2007) from where the carbon will be released to the atmosphere through subsequent decomposition. For this pool, we estimated an average C sink, for the entire period, equal to −43 Mt CO_2_ year^−1^. For the mineral soil we estimated a modest and rather stable C sink over the entire period, equal on average to −3 Mt CO_2_ year^−1^. This trend is consistent with increasing biomass of the EU forests, which means increasing inputs from litter and dead wood to the soil pool, and with the short time horizon considered, i.e., the process of soil C accumulation is typically a slow process. This process is simulated by the CBM through a series of biomass annual turnover rates and transfer rates [25]. Similarly to other soil models [43] the results provided by CBM may be influenced by uncertainty in the model initialization that may directly affect the estimate of the C stock change of this pool [44].

In terms of C stock change, we estimate average values, for the entire period, equal to 0.01, 0.08 and 0.60 Mg C ha^−1^ year^−1^ respectively for living biomass, DOM and soil.

The CO_2_ sink of the FM living biomass pool estimated by the CBM is about 12 % lower than the data reported in the EU GHGI^2^ (see Fig. 1, plot a) and is in line with most of other studies with similar area and time frames (e.g. [45, 46]). The largest differences with the GHGI, in 2000, 2005 and 2007, are related to the different assumptions about the impact of natural disturbances (mainly storms that occurred in central and northern European countries). Indeed, the effect of natural disturbances on the redistribution of C among pools (from living biomass to DOM), well represented in the CBM, is often not evident in the GHGIs [22, 26]. The difference between the CBM and the EU GHGI is reduced to 8 % when DOM and mineral soil are also considered. Since the uncertainty of CO_2_ estimates for “forest land remaining forest land” at the EU level is around 18 % [13],^3^ and given the fact that methods to estimate emissions/removals by the CBM are largely independent from those of the EU countries [26], we consider the match between the CBM results and the EU GHGI to be satisfactory. The increasing discrepancy in more recent years is mainly due to few countries (mainly Poland and France), which will deserve further investigation, e.g. inappropriate data or assumption used by the CBM model or problems with the GHGIs. Indeed, at country level, where updated NFI data are available and the model’s assumptions on harvest and natural disturbances are consistent with the countries’ input data, the estimates provided by the CBM are generally consistent (both in the trend and in the amount) with the GHGI data [26].

For AR (Fig. 1, panel b), the living biomass C sink gradually increases until 2003, due to the constant annual rate of AR prior to 2008 (see Fig. 7 in the “Methods” section). We estimated a very small source for the atmosphere from DOM and soil, due to the effect of afforestation on the soil pool during the first years [47]. As explained in the methods, the biomass C sink is directly related to the values reported by the yield tables applied by model. In most cases, due to the young age of the forests that were afforested since 1990, we assumed that no silvicultural treatment was applied to broadleaved species stands younger than 15 years and to coniferous stands younger than 20 years. The only exception was for Portugal’s Eucalyptus plantations, where we assumed a minimum rotation length of 12 years. Due to the effect of these treatments, the biomass sink has a first step in 2003–2004 (due to the harvest on Eucalyptus plantations), and a second step in 2009–2010, due to the first harvest applied to coniferous plantations. Overall, the total amount of harvest provided by AR is negligible, equal to about 6.3 Mm^3^ in 2012, i.e., about 1.2 % of the total amount of harvest obtained from FM at the EU level. The total annual C sink (−54 Mt CO_2_ year^−1^) provided by AR for the CP1 (2008–2012) is equal to about 1.2 % of the total GHGs in the EU for the same period (without LULUCF).

For deforestation (Fig. 1, panel b), the CBM estimates the loss of C from the living biomass, DOM and soil pools based on the areas subject to deforestation (taken from countries’ GHGIs). Overall, for the CP1 (2008–2012), these emissions equal about 0.7 % of the total GHGs in the EU for the same period (without LULUCF).

Our estimates for AR and D compare very well with the EU GHGI for CP1 (2008–2012) [48]. While emissions from drained organic soils may be important in some MS (e.g. Finland, Sweden, Ireland) at the EU level the impact of organic soils for AR and D is small (about 2–3 Mt CO_2_ year^−1^). Overall, this good match with the GHGIs is expected, because the CBM uses the same rates of AR and D areas as reported in the GHGIs and because of the good agreement for the estimates of biomass carbon densities. For AR, however, a certain degree of independence between CBM and GHGIs arises from the choice of the yield tables and the harvest assumptions.

### Main drivers determining forest C dynamics

**Fig. 2:**
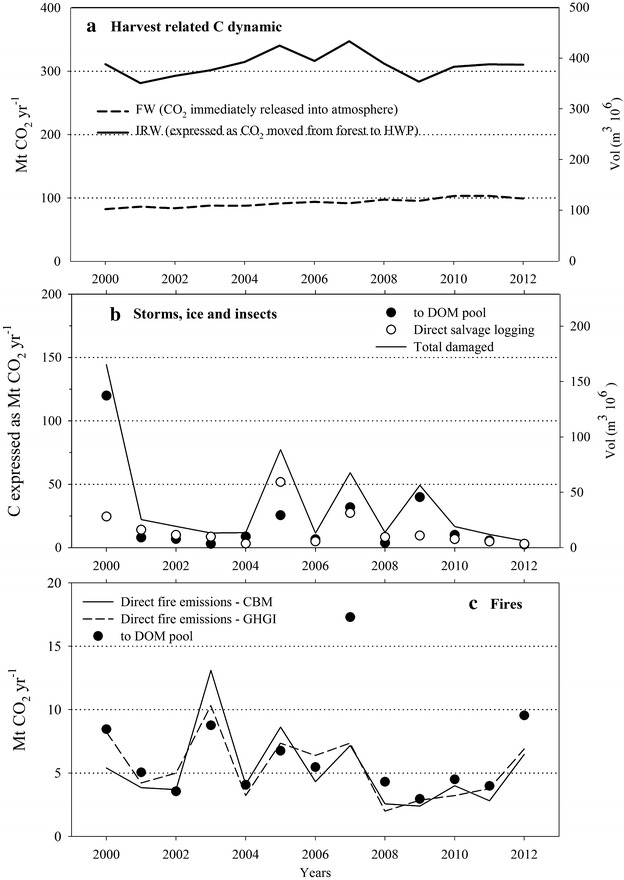
**a** Harvest-related C dynamics (in Mt CO_2_ year^−1^, reported on the *left axis*) and volume removed according to our model (in million m^3^, on the *right axis*), in terms of fuelwood (FW) immediately released into the atmosphere and industrial roundwood (IRW) moved from forest to HWP; **b** indirect total CO_2_ emissions (in Mt CO_2_ year^−1^, reported on the *left axis*) due to storm and insect attacks (reported as volume, in million m^3^, on the *left axis*), further distinguished between the amount of biomass moved from living biomass to DOM pool (*black dots*) due to the disturbances and directly recovered as salvage logging (*white dots*); **c** CO_2_ emissions (in Mt CO_2_ year^−1^) due to fires, distinguished between direct—i.e. immediately released into the atmosphere (according to our estimates and compared with the data reported in the EU GHGI [13])—and indirect emissions, i.e. moved from biomass to DOM pool (*black dots*), from where it will decompose in subsequent years

The forest C sink is essentially the difference between the net increment and the losses, i.e., harvest and natural disturbances. Forest growth, and the evolution of net annual increment^4^ over time is estimated during the model run, by combining, for each country and time step (i.e., year), a yield table library based on the NFI annual increment with the forest inventory and its age class distribution (see [25, 44]). Given that the net increment typically changes relatively slowly and in recent years appears rather stable at EU level [49] here we focus on harvest (Fig. 2, panel a) and natural disturbances, such as storms and ice (panel b) and fire (panel c).

#### Harvest

The CBM represents the amounts used for fuelwood (FW) and for industrial roundwood (IRW) (Fig. 2, panel a). According to the IPCC [11] and the UNFCCC [50], for the Second Commitment Period of the KP (KP-CP2) the C in the FW has to be accounted as a direct CO_2_ emission into the atmosphere, while the C in the IRW products has to be further quantified to estimate the C stock changes in the HWP pool, including product categories. End-of life disposal of HWP in landfills is considered an instantaneous oxidation.

According to our estimates using the CBM model, the direct emissions related to the FW continuously increased, from about 82 Mt CO_2_ year^−1^ in 2000 to about 100 Mt CO_2_ year^−1^ in 2012 (i.e., +2 % year^−1^). Based on the country-specific assumptions applied by CBM, the FW may be provided by: (i) direct harvest removals, i.e., specific silvicultural treatments applied to forest stands (e.g., clearcuts on coppices or commercial thinnings on high forests); (ii) indirect harvest of branches, other wood components and snags during other silvicultural treatments (i.e., thinnings and clearcut where the merchantable biomass is used as IRW); and (iii) the salvage logging after disturbance events (mainly fires).

**Fig. 3:**
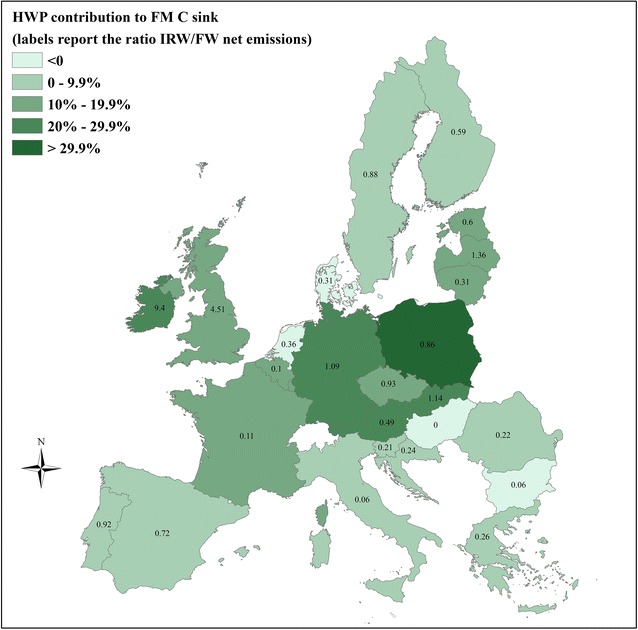
HWP mitigation contribution to the forest management (FM) C sink (*colors* as per legend). The numbers report the ratio between the industrial roundwood (IRW) C sink and the fuelwood (FW) direct emissions to the atmosphere

The amount of IRW that was moved from living biomass to the HWP pool was equal, on average, to 309 Mt CO_2_ year^−1^ between 2000 and 2012. The two peaks reported in 2005 and 2007 are due to salvage logging after the storms in 2005 and 2007 (see Fig. 2, panel b). Interestingly, no major peak is reported by the statistics after the big storm that occurred in 1999/2000 and after the storms occurred in some countries (i.e., Austria, Estonia and, above all, France) in 2009, suggesting that salvage logging either was not very relevant or it was spread over more years. According to Rüter [51], the analysis of IRW data to obtain C stock changes in the HWP pools involves the service life of products (i.e., the annual decay rate), the estimate of the domestic production, the balance between C inflow and outflow from the HWP pool, the exclusion of harvest from deforestation, and many other factors. Taking into account all these factors and applying the same harvest rate used in the present study, Pilli et al. [30] have applied the IPCC Tier 2 production approach [11] to estimate the HWP mitigation potential at the EU level. The resulting net CO_2_ sink in the HWP pool is equal on average to −44 Mt CO_2_ year^−1^ between 2000 and 2012. The ratio between the IRW C sink and the FW direct emissions to the atmosphere (on average 93 Mt CO_2_ year^−1^ between 2000 and 2012) is equal, on average, to 0.48 at EU level but it varies between countries, as highlighted by the labels in Fig. 3.

The sum of the net CO_2_ emissions of the forest pools plus the HWP (as estimated by [30]) is, on average, equal to −409 Mt CO_2_ year^−1^ between 2000 and 2012 (Fig. 1, panel c). Our estimates on HWP at the EU level are very similar to the data submitted by the countries to the KP [52], and indicate that the HWP mitigation contribution is currently equal to about 10 % of the total forest net CO_2_ emissions at the EU level. Pan et al. [53] estimated that at the global level, the C sequestration in HWP accounted for 8 % of the total C sink in established forests. As expected, at the EU level this percentage is higher and in five out of 26 countries the contribution of the HWP pool to the total FM C sink is >20 %, for the historical period 2000–2012 (see Fig. 3). However, because these estimates are based on the IPCC production approach, the C sink in HWP for countries with large exports is attributed to the country of harvest, i.e. where the wood originated and which may not be the country where the wood is in use.

As highlighted by Pilli et al. [30], in the future, the current HWP sink can be maintained either by further increasing (on average by 1 % per year) the current harvest, or by shifting more of the harvest to long-lived products [54]. In some countries, this contribution is negligible compared to the total forest C sink. In four countries where the IRW pool is a C source, the HWP pool has negative impacts on the overall C sink. This highlights the need to consider the specific national circumstances, when analyzing the possible contribution of the HWP C pool as potential mitigation tool.

#### Natural disturbances

##### Storms and ice

**Fig. 4:**
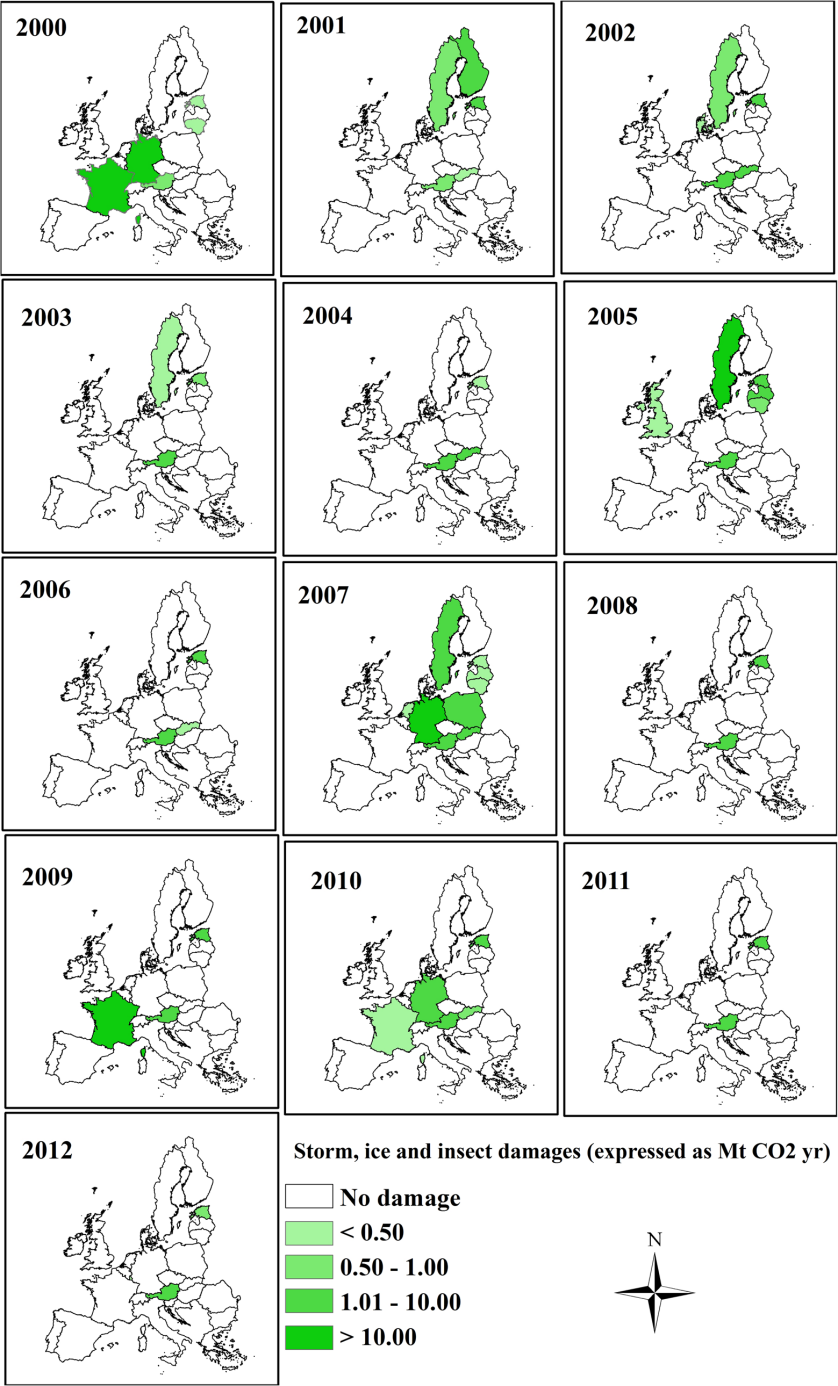
The map reports, for each year and country, the amount of living biomass C stock (expressed as Mt CO_2_ year^−1^, even if these are not direct CO_2_ emissions to the atmosphere) damaged by storm, ice and insect disturbances, as estimated by our model

**Fig. 5:**
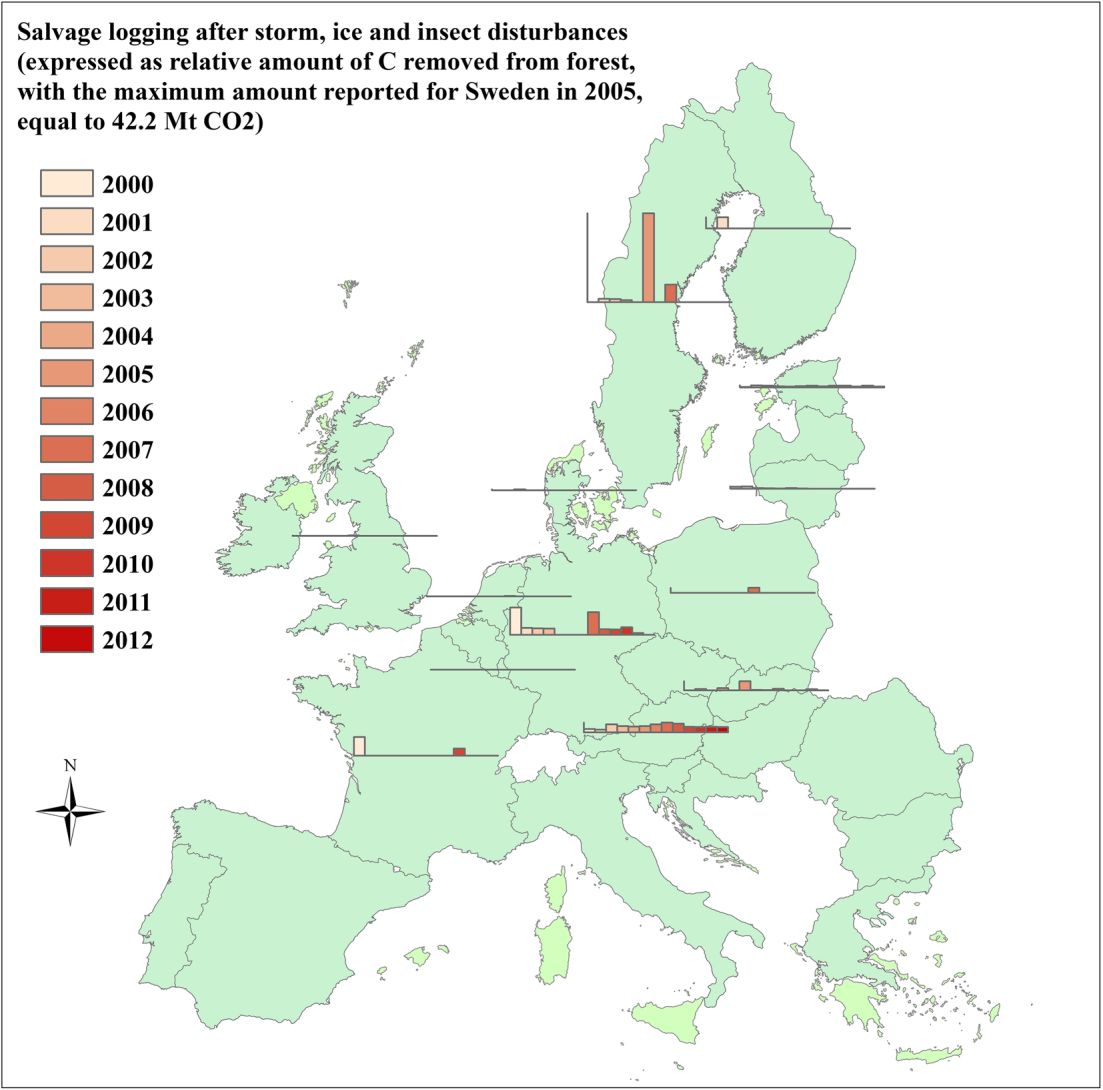
Salvage logging after storms, ice and insect disturbances, as estimated by the CBM between 2000 and 2012, reported as relative amount of C removed from forest, with the maximum amount reported for Sweden (2005) equal to 42.2 Mt CO_2_

The overall C dynamics related to storms and ice are shown in Fig. 2, panel B. These disturbances do not produce any direct emission of CO_2_ to the atmosphere, but they cause a transfer of C from the living biomass to both the DOM pool and the HWP pool (due to direct salvage logging). This process, reported in detail at the country level in Fig. 4, is simulated by the CBM through disturbance matrices for each disturbance type applied to each country (according to available information from the literature). Disturbance matrices quantify the proportion of C that is moved: (i) from the living biomass to DOM and (ii) from the living biomass to the HWP pool (see Fig. 5). Indeed, a consistent fraction of the living biomass damaged by these events is removed as salvage logging immediately after the disturbance (i.e., in the same year) or few years later (see [26, 55, 56]). The storm that occurred in 1999/2000 (reported as 2000 in Figs. 2, 4 and 5), with a total C stock transfer from the living biomass to DOM of about 150 Mt CO_2_, caused opposing peaks in the living biomass and DOM pools (Fig. 1, panel a). The same effect is clearly reported for the other major disturbances (e.g., 2005, 2007 and 2009). On average, we estimated that, between 2000 and 2012, about 34 Mt CO_2_ year^−1^ were moved from the living biomass to DOM and to HWP due to the effects of storms; excluding the amount of biomass directly removed as salvage logging, this amount decreases to 21 Mt CO_2_ year^−1^.

The main storms at the EU level (the area affected by ice is negligible compared with storms) occurred in 1999/2000 and 2005 (see Figs. 2, 8, panel b in the Material). In the first case (which we count as 2000), the so called storms “Lothar” and “Martin”, occurred on 27th–28th December 1999, and affected mainly France and Germany (Fig. 4). According to Gardiner et al. ([55], Appendix 3) between 184 and 204 Mm^3^ were directly damaged by these events. Based on our model run, about 170 Mm^3^ were damaged. In 2005, different storms affected many European countries, including “Gudrun” and “Erwin”, which damaged about 75 million m^3^ in northern Europe (see [55], Appendix 3).

Overall, between 2000 and 2012, about 272,000 ha year^−1^ (based on the data collected by our study) were affected by storms in EU countries and, according to our estimates, on average 36.5 Mm^3^ year^−1^ were damaged. The direct salvage of storm residues simulated by CBM was equal, on average, to 13 Mm^3^ year^−1^ but with high interannual variability (see Fig. 5). Indeed, when large disturbances occur, we cannot expect that all the biomass affected by storms will be removed during the same year (this is the case for the two main disturbances that occurred in 1999–2000 and 2005). A fraction of this biomass will be removed during the following years (see for example the case of Germany in Fig. 5 between 2000 and 2003). A further amount of salvage logging may be recovered through the normal silvicultural practices (i.e., thinnings, clear-cuts, etc.) applied by the model at the country level but this amount cannot be directly estimated. On average, excluding the largest disturbance events (i.e., 2000 and 2005), we estimated that about 40 % of the total biomass damaged by storms (including branches and other wood components) was directly removed as salvage logging between 2000 and 2012.

##### Fire

**Fig. 6:**
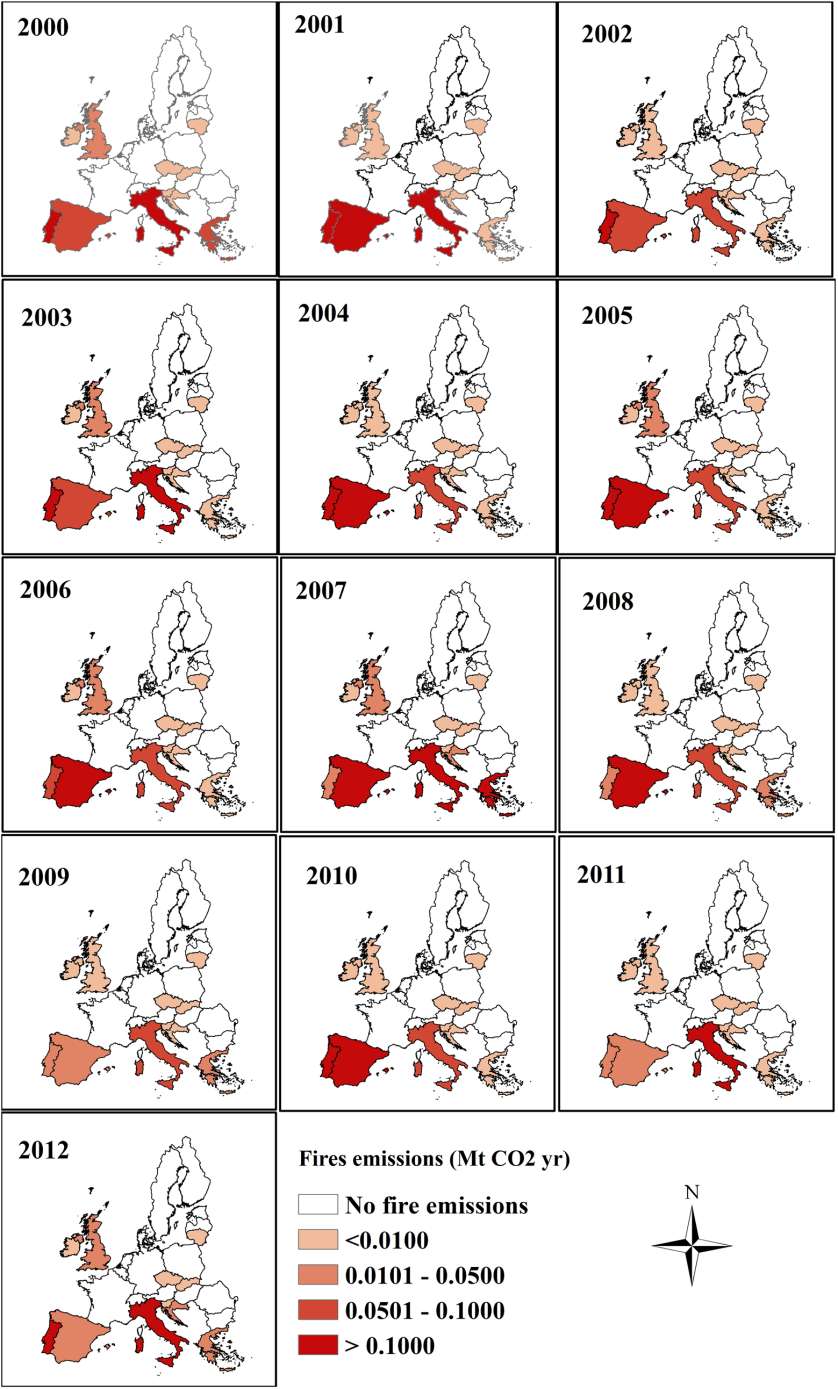
The map reports, for each year and country, the direct CO_2_ emissions to the atmosphere (in Mt CO_2_ year^−1^) due to fire disturbances, as estimated by our model. Where no direct fire emissions is reported, they were assumed as negligible, according to the information reported by each country on the National Inventory Reports submitted to UNFCCC

The second major disturbance type considered by our study is wildfire and slash burning (if relevant). Average annual direct emissions to the atmosphere due to the burning of biomass and dead organic matter are equal to 5.27 Mt CO_2_ year^−1^ between 2000 and 2012 (Fig. 2, panel c). As for the storms, strong inter-annual variations are related to the amount of area burned with peaks in 2003, 2005 and 2007, mainly concentrated on the Mediterranean countries (see Figs. 6, 8, panel c). In some cases (Slovak Republic and Lithuania), the emissions reported in Fig. 6 are mainly due to burning of harvesting residues.

Based on our assumptions, the total emissions due to fires are equal, on average, to 15 % of the total indirect losses due to the effect of storms. Indeed, while storms have a clear effect on the forest living biomass pool (Fig. 1, panel a), the effect of fires on this pool appears, at the EU level, much less evident. However, while storms do not contribute to direct CO_2_ emissions and a considerable amount of wood is recovered through salvage logging, fires do cause direct emissions to the atmosphere and the amount of salvage logging varies considerably among countries. For example, in Portugal a substantial amount of wood may be recovered as salvage logging after fire disturbances, while this practice is negligible in Greece and Italy [26]. This explains the peak on transfers of living biomass to DOM reported in 2007 (see Fig. 2, panel c), when fires mainly occurred in Italy and Greece. Overall, our estimates are consistent with the total CO_2_ emissions reported in the EU GHGI, except for few a years (e.g. 2000, 2003 and 2007). A full comparison between the CBM and the EU GHGI however is difficult because of the different assumptions and methodologies used by some MS (see [13, 14]).

## Conclusions

This paper analyzed the CBM results at the EU level, in terms of C emissions and removals for all forest activities foreseen by the Kyoto Protocol: forest management (i.e. land in the forest land-use category in 1989), afforestation/reforestation and deforestation (, i.e. forest related land-use changes since 1990). We considered all forest carbon pools (including HWP for FM) and we analyzed the main drivers of forest C stock and sink dynamics over the period 2000–2012 (i.e., harvest, natural disturbances and land-use changes).

Overall, the sink estimated by the CBM from FM, AR and D during 2000–2012 corresponds to about 7 % of total GHGs at the EU level for the same period. The sink from the HWP pool contributes an additional 1 %. The CBM results for FM, AR and D are very close to the reported values from the EU GHGI, i.e. 8 % lower for FM (in the CBM vs. GHGI) and almost identical for AR and D.

In absolute terms, the impact of harvest is much greater than natural disturbances but, because of salvage logging, the impact of natural disturbances is often not easily distinguishable from the impact of harvest. Over the period analyzed in our study, the sum of these two drivers often caused year-by-year variations of about 10–15 % of the FM sink at the EU level. Between 2000 and 2012, land-use changes are also important: the annual sink from AR reached 15 % of the FM sink, while annual emissions from D were on average equal to nearly 10 % of the FM sink.

Our analysis on natural disturbances showed that at the EU level the impact of storms on the C stock balance is quantitatively far more important than the impact of fires, i.e. 5–10 times greater when both direct and indirect emissions are considered. While other studies quantified the impact of these events on the European forest carbon balance (e.g. [23, 57]), the detailed analysis of the C dynamics of natural disturbances presented in this study is useful both to identify climate change mitigation options and to allow better process understanding for the purpose of accounting emissions and removals under the KP and the post-2020 EU regulations. In particular, while storms cause a transfer of C from biomass to DOM pools (from where it will decompose unless salvage logged), fires cause an approximately equal amount of direct emission (of CO_2_ to the atmosphere) and indirect emission (i.e. C moved from living biomass to DOM from where it will decompose). A consistent fraction of the total amount of harvest may be provided by salvage logging after disturbance events, and in several cases the impact of natural disturbances is not visible in the GHGIs either because the dynamics described above are not taken into account (e.g. Italy, [44]) or because the methods used to estimate GHGs do not detect interannual variations (e.g. Germany, [26]). In the light of the rules under the KP [11], our results suggest that: (i) different management strategies (e.g., salvage logging) applied after natural disturbances may significantly affect the total C balance, and (ii) modelling the detailed C dynamics associated to natural disturbances is an essential prerequisite to apply the provision of natural disturbances under the KP (i.e. excluding the corresponding emissions and subsequent removals from the accounting under certain circumstances).

In conclusion, this study presents the application of a consistent methodological approach, based on an inventory-based model, adapted to the forest management conditions of 26 different EU countries. The approach captures, with satisfactory detail, the C sink reported in the EU GHGI and the country-specific variability due to harvest, natural disturbances and land-use changes. These results provide the basis for possible policy-relevant future applications of the CBM, e.g., as a tool to support GHGIs (e.g. on accounting for natural disturbances) and to verify GHGIs, and for the simulation of specific scenarios at EU level. Applying the same model from a regional [58], to country [26, 44] and at the EU level (this study), may help a consistent assessment of different forest sector mitigation strategies appropriate to the specific regional circumstances, and in evaluating the overall contribution of the forest sector towards EU emissions reduction targets.

To our knowledge, this is the most comprehensive study of its kind at EU level. Indeed, even if the EU forest C sink was previously analyzed by many other studies, none of them provided an overall assessment disaggregated between FM, AR and D, including all the forest pools, HWP and natural disturbances, and a comparison with the EU GHGI. Such comprehensive assessments and comparisons are increasingly needed to help improving the quality of GHGIs and ultimately increasing the credibility of the forest sink as a potential mitigation option within the EU [27] and the international frameworks [10].

As part of an integrated modeling framework, further possible developments include linking the CBM to models predicting land-use changes, the impact of climate change on primary productivity, the harvest demand and the material substitution effects of the industrial roundwood products.

## Methods

This study used the Carbon Budget Model (CBM, [25]) to estimate forest C stock and the net CO_2_ emissions in 26 EU countries, covering a total forest area of about 146 Mha, disaggregated in 189 forest types (FTs) distributed over 178 administrative regions and 35 climatic units. The area included about 138 Mha of FM (at time step zero of the model runs) and, in 2012, 8 Mha of AR, and 2.8 Mha of D, since 1990. Due to the D area, the area of FM slightly decreased during the model runs, but this area decrease was compensated by an increase in AR area. Unproductive forests (according to countries’ GHGIs) and overseas territories were not included in this study [26].

The spatial framework applied by the CBM conceptually follows IPCC Reporting Method 1 [11] in which the spatial units are defined by their geographic boundaries and all forest stands are geographically referenced to a spatial unit (SPU). For FM, the Carbon Budget Model and the general methodological assumptions applied to each country were described previously [26]. Further details can be found elsewhere for the model itself (e.g. [25]), for its applications to European countries [26, 44] and for the representation of natural disturbances [26]. A summary of the main NFI input data used for each country is reported in Table 2. We considered 26 administrative units (i.e., European countries, as reported by Table 2) and 35 climatic units (CLUs, as defined by [59]), with mean annual temperatures (mainly affecting the DOM turnover rate), ranging from −7.5 to +17.5 °C.

**Table 2:** Summary of the main parameters applied by CBM model for each country

Country	Original NFI year	Time step 0 (years)	CBM FM area (Mha)^b^	Harvest rate (av. 2000–2012, Mm3)	County specific biomass equations
Austria	2008	1998	3.2	22.9	X
Belgium	1999	1999	0.7	4.3	
Bulgaria	2000	2000	3.2	5.3	
Croatia	2006^a^	1996	2.0	4.6	
Czech Republic	2000	2000	2.6	17.0	X
Denmark	2004	1994	0.5	2.3	
Estonia	2000	2000	2.1	7.9	
Finland	1999	1999	21.7	55.0	
France	2008	1998	14.6	54.9	
Germany	2002	1992	10.6	74.7	X
Greece	1992^a^	1992	1.2	1.6	
Hungary	2008	1998	1.6	6.2	X
Ireland	2005	1995	0.5	2.8	
Italy	2005	1995	7.4	10.2	X
Latvia	2009	1999	3.2	15.8	X
Lithuania	2006	1996	2.0	7.7	
Luxembourg	1999	1999	0.1	0.3	
Netherlands	1997	1997	0.3	1.2	
Poland	1993	1993	8.9	37.8	
Portugal	2005	1995	3.6	12.2	X
Romania	1985	1985	6.6	17.2	X
Slovakia	2000	2000	1.9	9.0	
Slovenia	2000	2000	1.1	3.3	
Spain	2002	1992	12.6	16.8	
Sweden	2006	1996	22.6	79.5	
United Kingdom	1997	1997	2.5	9.8	
EU			137.9	480.7	8 countries

The table reports the NFI original reference year; the year since the model was applied; the FM area used by CBM at time step 0; the average harvest rate used; the countries where specific equations to convert the merchantable volume into aboveground biomass were selected. Two countries were not modeled: Cyprus (no NFI data available) and Malta (very small forest area, mainly covered by shrub lands)

^a^Analysis based on data from forest management plans

^b^FM area used by CBM at time step 0. According to KP rules, FM is the area of forest in 1990, decreased with any subsequent deforestation. The FM area is taken from the official submissions made by countries to UNFCCC/Kyoto Protocol, giving priority to data from KP-CRF tables when available (i.e., if FM had been elected during the first KP commitment period), or alternatively taking data from the convention CRF tables (using ‘forest land remaining forest land’ in 1990 as a proxy for FM). To obtain FM area at time step 0, the D area reported by all countries under the Kyoto Protocol was used. Please note that CBM runs did not include forests reported as “not productive” (e.g., 0.4 Mha in Austria, 0.02 Mha in Bulgaria, 5 Mha in Sweden) and overseas territories (8.2 Mha in France)

Within a SPU, each forest stand is characterized by age, area and seven classifiers that provide administrative and ecological information, the link to the appropriate yield curves, and parameters defining the silvicultural system such as the forest composition (defined according to different FTs), the management type (MT), and the main use of the harvest provided by each SPU, as fuelwood or industrial roundwood. For each country, these parameters were mainly derived by national forest inventories. According to country-specific information, MTs may include even-aged high forests, uneven-aged high forests, coppices and specific sylvicutural systems such as clear-cuts (with different rotation lengths for each FT), thinnings, shelterwood systems, partial cuttings, etc. (detailed information on the main model’s assumptions for five representative countries are reported in the Supplementary Information of [26]).

To assess the FM area, data from KP reporting were used when available (for 18 countries in the period 2008–2012); alternatively “forest land remaining forest land” data (from UNFCCC reporting) were used, i.e. for all countries before 2008 and for those counties that did not elect FM during 2008–2012. Countries’ data for AR and D for 2008–2012 always came from KP reporting.

The following carbon pools were considered: living biomass (aboveground and belowground), dead organic matter (DOM), mineral soil and harvested wood products (HWP). Even if the CBM estimates CH_4_ and N_2_O emissions, this study includes only CO_2_ and excludes organic soils.

In the CBM, species-specific, stand-level equations [60] convert merchantable volume production into aboveground biomass, partitioned into merchantable stemwood, other (tops, branches, sub-merchantable size trees) and foliage components. Where additional information provided by NFIs or by literature was available (see last column in Table 2), country-specific equations were selected to convert the merchantable volume into aboveground biomass.

The CBM starts the initialization process with all DOM pools containing zero C stocks and then simulates multiple iterations of growth and stand-replacing disturbances, gradually increasing the size of the DOM pools [25]. The rotations continue until the slowly-decaying C pools at the end of two successive rotations meet a difference tolerance of 0.1 %. Once this criterion has been met, the CBM applies a user-selected last disturbance event which affects the amount of C in the DOM pools, and then links DOM dynamics to biomass dynamics. Inputs from biomass to DOM pools, during the model run, result from biomass litterfall and turnover as well as natural and human-caused disturbances. The DOM parameters were first calibrated and validated on some specific study at country and regional level [44, 58] and, if necessary, further modified for specific countries [26].

We use two sets of yield tables (YT) in these analyses [26, 44]. Historical YTs derived from the standing volumes per age class reported by the NFI represent the impacts of growth and partial disturbances during stand development. Current YTs derived from the current annual increment reported in country NFIs represent the stand-level volume accumulation in the absence of natural disturbances and management practices.

For 21 countries, we also evaluated the impact of natural disturbance events (a summary at EU level is reported in Fig. 8, below), including storms and ice (15 countries), fires (11 countries) and insect attacks (i.e., bark beetles attacks, for 2 countries). Specific information on the assumptions on natural disturbances are reported by [26],

The following sections provide specific information on the application of CBM to areas affected by land-use changes (AR, D lands) and a summary, at the EU level, of the main assumptions on harvest applied by our study.

### AR and D assumptions

**Fig. 7:**
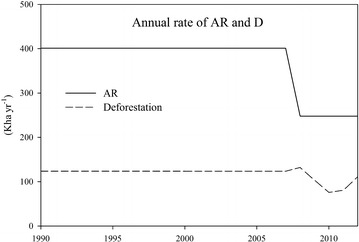
Total annual rate (kha year^−1^) of afforestation (AR) and deforestation (D), used by CBM at the EU level, based on countries’ GHGIs under the Kyoto Protocol. Data from 1990 to 2007 are the cumulative area of AR and D in 2008 converted to annual average rates of land-use change

We used for the analyses of AR and D areas the same 26 administrative units (i.e., European countries) and 35 CLUs applied for FM. For both AR and D, we used the area reported up to 2012 by each country in the GHGIs submitted the KP (Fig. 7). Before 2008, only the cumulative values since 1990 are available [13] and therefore we used an average annual rate of AR and D for the period 1990–2007.

AR was modelled through country-specific model runs, always starting in 1990. The total amount of AR per year was distributed between different FTs using the same proportions of FTs observed in the FM area. Based on a preliminary model assessment, we generally used the current YT library for AR [61]. This library was derived from the increment data reported by each country. These values represent the gross volume yield of each stand (while the YTs derived by the standing volume include the impact of past silvicultural treatment) and therefore are more suitable for young stands, generally younger than 20 years (i.e. for AR), where in general no silvicultural treatments are applied.

We assumed that the harvest rate was entirely satisfied by the FM area (with Portugal the only exception, see [26]), apart from a modest amount of harvest provided by D. The possible amount of harvest provided by AR (i.e. post-1990 forest) was generally very small [61].

For AR we estimated the maximum potential (and theoretical) amount of harvest provided assuming a common set of silvicultural practices for all the countries, with a 15 % commercial thinning applied to broadleaved forests 15-years or older and a 20 % commercial thinning applied to coniferous forests 20-years or older [61]. The only case where the harvest from AR was relevant was Portugal.

### EU summary of the main input data

**Fig. 8:**
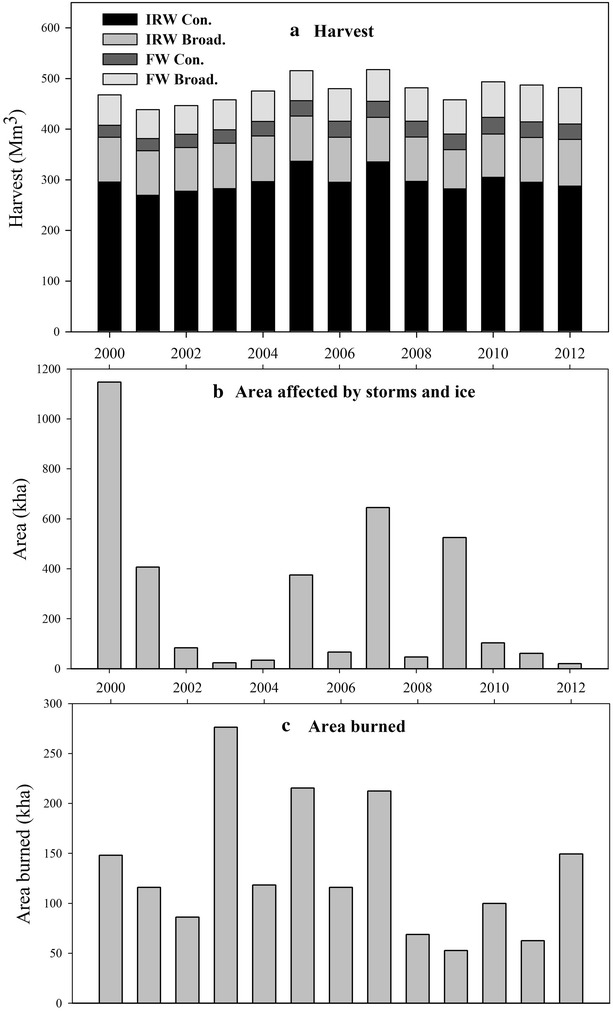
**a** Total harvest demand (in millions m^3^) applied by CBM at the EU level, further distinguished between industrial roundwood (IRW) and fuelwood (FW) and between coniferous and broadleaved species; **b** total area affected by storms and ice (in kha); **c** total area affected by fire (in kha)

A summary, at the EU level, of the main input data applied by our study is reported in Fig. 8, including: the total harvest rate applied by CBM at the EU level, further distinguished between industrial roundwood (IRW) and fuelwood (FW) and between coniferous and broadleaved species (panel a); total area affected by storms and ice (panel b); total area affected by fire (panel c). The harvest rate was mainly derived by a specific study on the HWP at EU level [30] where, for each country, FAOSTAT data (further distinguished between IRW and FW) were collected, compared with other data sources (i.e., Forest Resource Assessment, National Inventory Report, NFIs, ecc.) and eventually corrected in order to account for possible inconsistencies (i.e., due to the bark fraction or different methodological approaches).

Since the amount of IRW used in the present study is consistent with [30], we derived the IRW C sink by this last study, based on the IPCC production approach [11]. In this method, estimates of net emissions are derived from a stock change calculations applied to products derived from domestic harvest, i.e., imported HWP are excluded in the national estimates.

## Abbreviations

AR: afforestation and reforestation
C: carbon
CBM: Carbon Budget Model
CP1: First Commitment Period
CP2: Second Commitment Period
D: deforestation
Dbh: diameter at breast height
DOM: dead organic matter
EU: European Union
FM: forest management
FT: forest type
FW: fuelwood
GHG: greenhouse gas
GHGI: greenhouse gas inventory
HWP: harvested wood product
IPCC: Intergovernmental Panel on Climate Change
IRW: industrial Roundwood
KP: Kyoto Protocol
LULUCF: land Use, Land-Use Change and Forestry
MT: management type
NFI: National Forest Inventory
NIR: National Inventory Report
UNFCCC: United Nations Framework Convention on Climate Change
YT: yield table

## Footnotes

1 Since this paper reports the results of a long-term study, based on data available until 2014, further comparisons with the data reported by the 2015 GHGI is not appropriate.

2 In the area-based comparison, the FM area of the EU GHGI was decreased by the same amount decreased for CBM runs, i.e. excluding unproductive forests and overseas territories, see “Methods” for further details.

3 The uncertainty raises to 25–50 % when analyzed at country level.

4 According to FAO, the net annual increment is defined as the average periodic increment of volume of all trees measured at a certain minimum Dbh, including the increment of trees which have been felled and excluding the increment of trees which have died during the reference period [62].
